# 3 Leadless Pacemaker Implantations in an 81-Year-Old Woman With a History of Transvenous Pacemaker Infection

**DOI:** 10.1016/j.jaccas.2024.102660

**Published:** 2024-11-20

**Authors:** Hiroto Yagasaki, Takeki Suzuki, Shunichiro Warita, Toshiyuki Noda

**Affiliations:** a Department of Cardiology, Gifu Prefectural General Medical Center, Gifu City, Japan; b Department of Medicine, Indiana University School of Medicine, Indianapolis, Indiana, USA

**Keywords:** cardiac implantable electronic device extraction, chronic atrial fibrillation, complete heart block, device dislodgment, leadless pacemaker, transvenous pacemaker infection

## Abstract

Leadless pacemakers (LPMs) offer an alternative for patients with challenging venous access or device infection history. Management of LPM battery depletion in frail patients presents unique challenges. We present the case of an 81-year-old frail woman with obstructive hypertrophic cardiomyopathy and complete heart block, previously treated with percutaneous transseptal myocardial ablation and a transvenous pacemaker, who received an LPM after device extraction for infection. On battery depletion, a second LPM was implanted but dislodged, thus necessitating extraction attempts. Given the high extraction risks, a third LPM was successfully implanted. This case highlights the feasibility of multiple LPM implantations in complex cardiac patients and demonstrates that a third LPM can be a viable option when extraction risks are high. This approach expands management options for frail patients with complex cardiac histories who are unsuitable candidates for traditional pacing systems.

## Graphical Abstract

**Figure:**
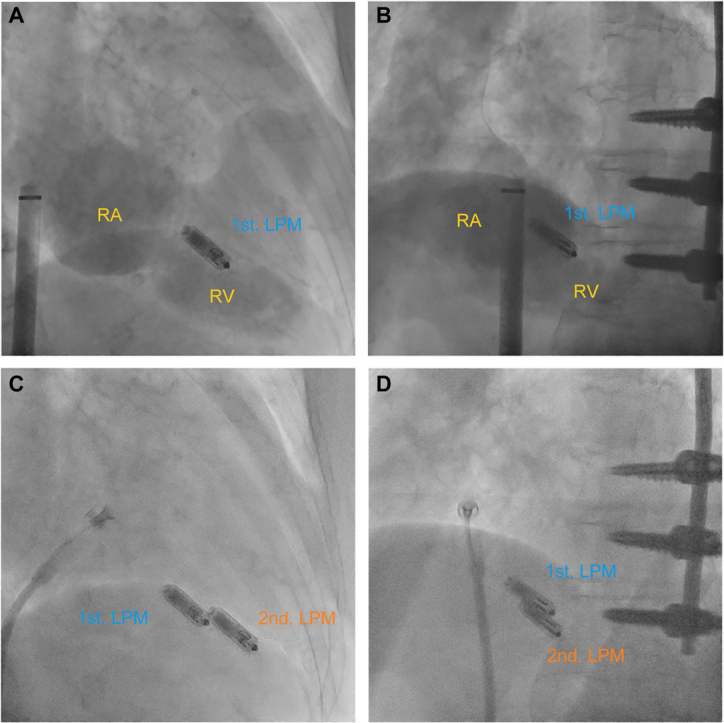

## History of Presentation

An 81-year-old frail woman with obstructive hypertrophic cardiomyopathy, chronic atrial fibrillation, and complete heart block, treated with percutaneous transseptal myocardial ablation (PTSMA) and a leadless pacemaker (LPM) (Micra VR, Medtronic), presented with pacemaker battery depletion. Her medical history included obstructive hypertrophic cardiomyopathy diagnosed 13 years earlier, PTSMA 10 years earlier, and transvenous pacemaker (TVPM) implantation for sick sinus syndrome 9 years earlier, later complicated by chronic atrial fibrillation and complete atrioventricular (AV) block. Three years previously, refractory bacteremia with spondylodiscitis led to device extraction and thoracic spinal fusion, prompting her first LPM implantation. Her clinical frailty score was 6, indicating that she needed assistance with all outdoor activities and minimal indoor activities.^1^

**Take-Home Messages**

• LPMs can provide a viable option for multiple reimplantations in patients with complex cardiac histories and a high risk of device infections.
• When faced with LPM complications, consider patient-specific factors and potential risks before deciding among extraction, reimplantation, or alternative pacing strategies.

## Past Medical History

She had a history of chronic diastolic heart failure, scleroderma, rheumatoid arthritis, pulmonary hypertension, and type 2 diabetes mellitus, in addition to the foregoing history.

## Differential Diagnosis

A probable cause of the early battery depletion was the high pacing threshold in the septal region, which presumably resulted from PTSMA.

The initial pacing threshold for the first LPM device was 2.88 V at 0.24 milliseconds with an impedance of 560 Ω. Following implantation, the threshold was unstable, fluctuating between 1.0 V at 0.24 milliseconds and 3.25 V at 0.4 milliseconds. The threshold stabilized after approximately 1 month, settling at 3.25 V at 0.24 milliseconds. The impedance remained relatively stable, ranging from 500 to 620 Ω throughout this period.

## Investigations

**Figure 1:**
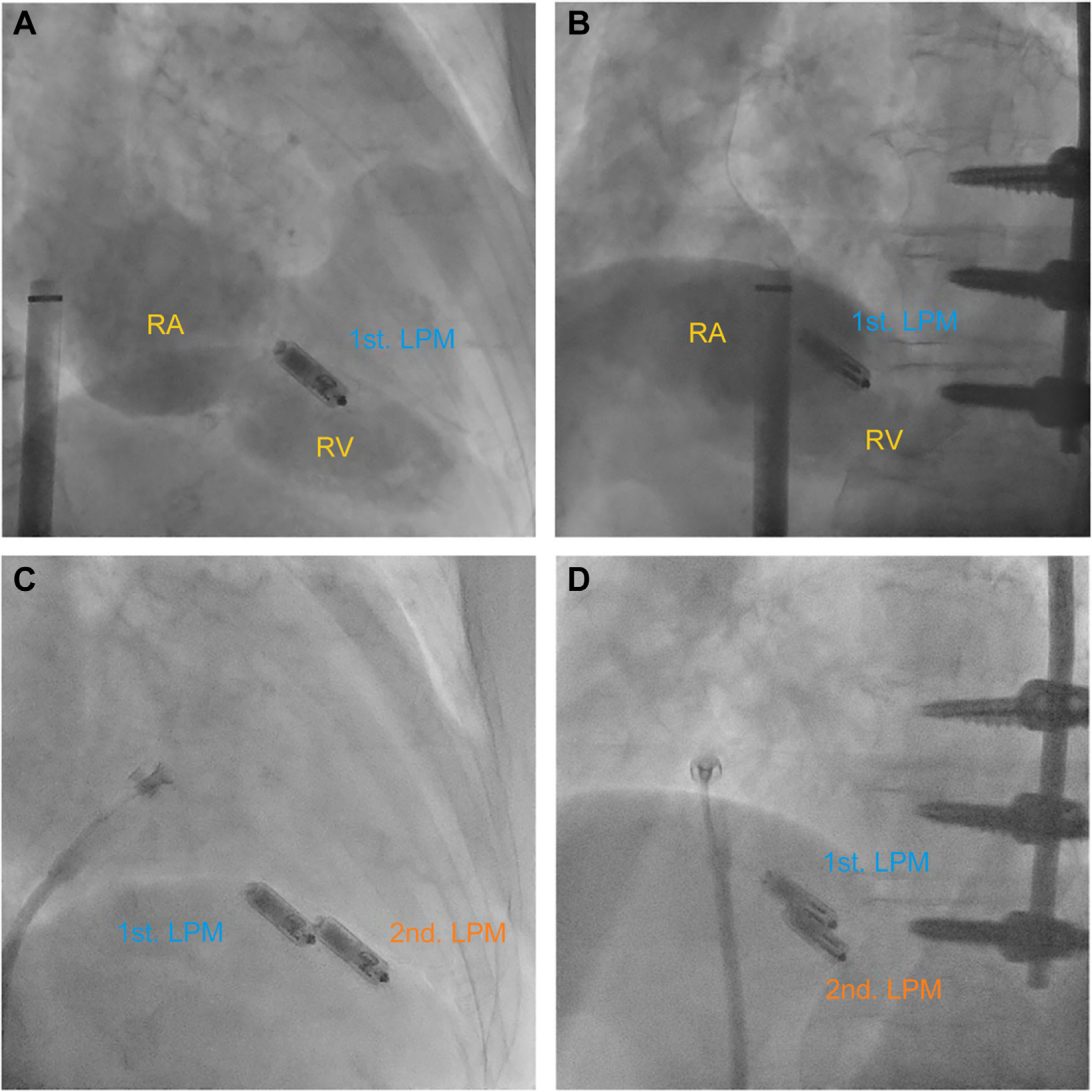
Second LPM Implantation (A and B) Right ventricular contrast and (C and D) the second leadless pacemaker (LPM) implantation at a 30° right anterior position and a 50° left anterior position. RA = right atrium; RV = right ventricle.

**Figure 2:**
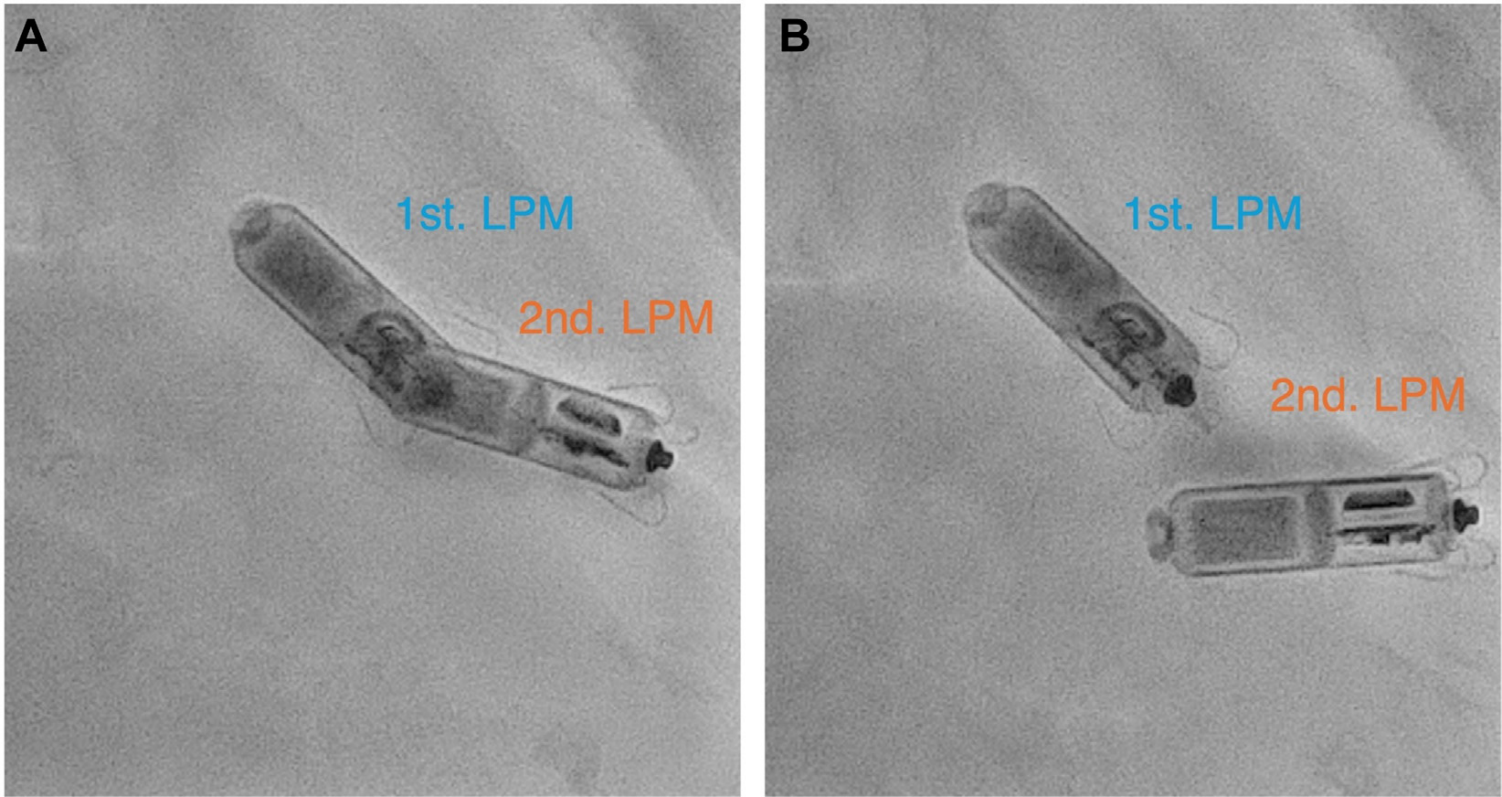
During and After Tether Removal (A) Initial stage of tether removal, showing parallel alignment of the first and second leadless pacemakers (LPMs). (B) During tether removal, the second leadless pacemaker exhibits increased mobility, assuming a nearly horizontal position on the screen.

Considering numerous underlying medical conditions that made her skin extremely fragile, including long-term steroid use and a history of device infection, a second LPM implantation was planned. We opted for the Micra AV (Medtronic) model to restore AV synchrony and because of its slightly longer battery longevity. Because of poor pacing thresholds previously noted in the midseptal region, likely from previous PTSMA, we targeted the lower septum (Figures 1A to 1D, Video 1). The initial pacing threshold was 2.0 V at 0.24 milliseconds, which was considered acceptable on the basis of her history. To ensure proper device fixation, 2 pull-and-hold tests were performed. The device appeared to be adequately attached to the septum, and we proceeded to cut the tether. However, during tether removal, the LPM’s mobility gradually increased and developed loss of capture with maximum output, with resulting bradycardia (Figures 2A and 2B, Video 2). This finding suggested possible microdislodgment or entanglement into right ventricular trabeculae that likely occurred during the tether cutting process.

## Management

We attempted extraction of the newly implanted LPM (second LPM). To extract the second LPM, an 8-F steerable sheath (Large-curl Agilis, Abbott) was inserted through the right femoral vein through a 23-F Micra Introducer Sheath (Medtronic) and a 6-F EN Snare device with a size of 9 to 15 mm (Merit Medical) was inserted through the steerable sheath.

**Figure 3:**
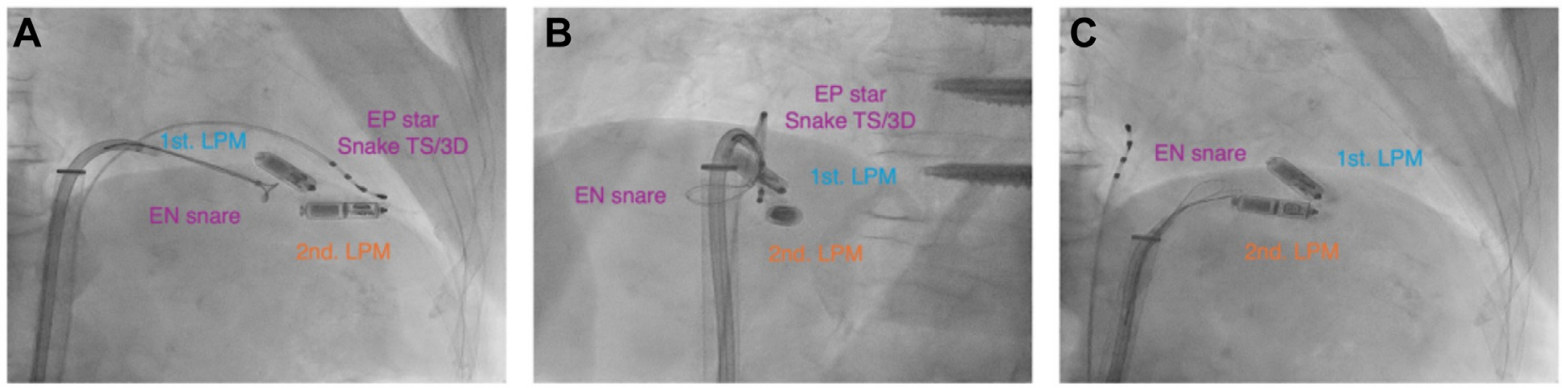
Extraction of the Second LPM (A and B) Use of the EN Snare (Merit Medical) and EPstar Snake (Japan Lifeline) at (A) a 30° right anterior position and (B) a 50° left anterior position. (C) The second leadless pacemaker (LPM) was drawn forward, creating a space for the third leadless pacemaker implantation.

Interference from the first LPM allowed the tip of the second LPM to retract only toward the inferior septal wall. Consequently, a 6-F sheath was inserted into the left femoral vein to introduce a steerable diagnostic electrophysiology catheter (EPstar Snake TS/3D, Japan Lifeline) for manipulating the second LPM. Using this catheter, we repositioned the second LPM for snaring. Despite these efforts, the attempt at capture was unsuccessful because the EN snare could not reach the LPM (Figures 3A and 3B).

We substituted a 6-F Mach 1 FR coronary guide catheter (Boston Scientific) for the outer sheath and successfully grasped the LPM by using the reintroduced EN snare. The second LPM was then pulled back (Figure 3C, Video 3); however, strong resistance was encountered during the pull-back. Because of potential cardiac injury risks including perforation, further pulling was deemed risky.

**Figure 4:**
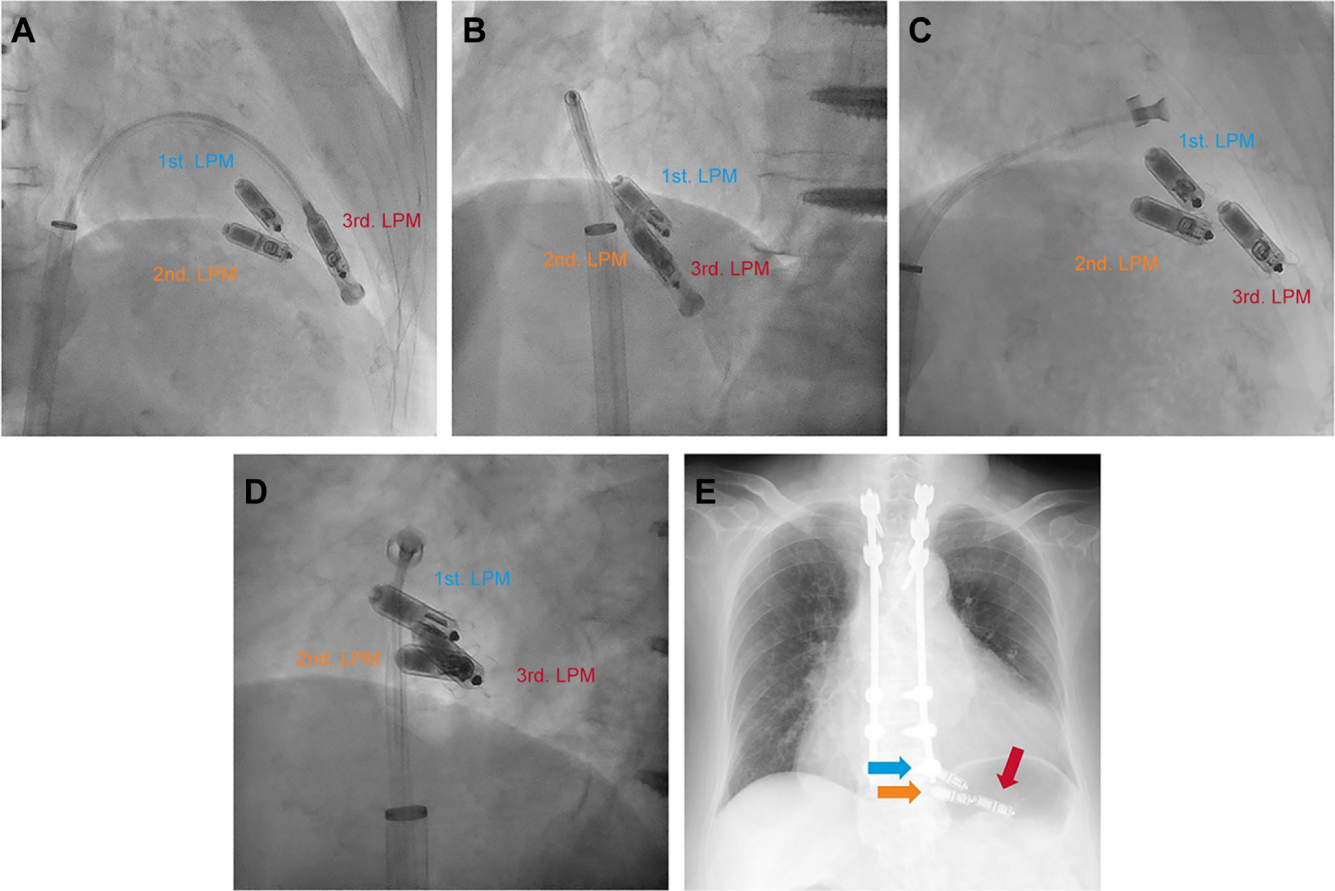
Third LPM Implantation Imaging (A and B) before and (C and D) after cutting the tether at 30° right and 50° left anterior positions. (E) Chest radiography showing the first, second, and third leadless pacemaker (LPMs), indicated by blue, orange, and red arrows, respectively.

Considering the patient’s history of device infection and compromised skin condition, along with the potential cardiac injury from retrieval, we opted to implant a third LPM (Micra AV). It was successfully placed in the apicoseptal position with a pacing threshold of 0.38 V at 0.24 milliseconds and an impedance of 830 Ω (Figures 4A and 4B, Video 4). The fixation integrity was ascertained with pull-hold tests, and the tether was removed without problems (Figures 4C and 4D, Video 4).

## Discussion

LPMs are beneficial for patients with challenging venous access or a history of cardiac implantable electronic device infection.^2^^,^^3^ This patient was well suited for LPM implantation because of her frailty, fragile skin, and long-term steroid use.^4^

The first LPM’s battery depleted approximately 3 years after implantation, likely hastened by a high pacing threshold from the myocardial damage caused by PTSMA, as well as pacemaker dependency. In such cases, particularly in younger patients or those highly dependent on pacemakers with elevated pacing thresholds, a second LPM implantation may be necessary.

The choice of LPM model for subsequent implantations required careful consideration. Although the Micra VR would have been sufficient for the patient’s condition (chronic atrial fibrillation and complete heart block), we opted for the Micra AV to restore AV synchrony and because of its slightly longer battery life. This decision was crucial given the patient’s history of high pacing thresholds. Even a marginal increase in battery longevity was deemed beneficial in this complex case.

Our experience with this case also revealed important technical considerations for multiple LPM implantations. When implanting a second or subsequent LPM, the presence of previously implanted devices could obscure clear visualization of the new device’s tines. This issue was evident in our case, where the left anterior oblique view overlapped with the first LPM (Figure 1D), thus making it challenging to confirm proper tine engagement and to assess device fixation in relation to the septum. Using multiple fluoroscopic angles and/or possibly intracardiac echocardiography could mitigate this shortcoming. Additionally, carefully performing and interpreting multiple pull-hold tests would be needed to ensure secure fixation.

Despite these precautions, the second LPM ultimately became dislodged, necessitating extraction attempts. As presented in this case, when the second LPM is dislodged, its extraction should be considered first because it is a new implantation. Some studies have reported successful LPM extractions at 5 to 7 years post-implantation.^5^^,^^6^ Furthermore, among 113 cases, successful LPM extraction was reported in all Micra recipients and in 90% of Nanostim (Abbott) recipients, within weeks to months of device implantation. However, 2 Nanostim recipients (3%) developed serious complications.^7^

On the basis of previous reports,^8^, ^9^, ^10^ we attempted extraction by using a snare and a guiding catheter and successfully grasped the device. However, we had to discontinue the extraction because of considerable resistance and potential interference from the first device and its surrounding capsule. During the procedure, transthoracic echocardiography revealed no apparent cardiac mechanical complications, tricuspid valve injury, or interference between the 2 devices.

Although we considered extracting the first LPM, the potential interference between the first and second LPMs and anticipated difficulties led us decide against it. Alternatives, such as TVPM or epicardial pacing, were also considered but eventually not pursued because of the patient’s history of frailty, previous device infection, and fragile skin condition. Because the pacemaker had reached the elective replacement indicator status, implanting a new device was deemed essential, resulting in a third implantation.

## Follow-Up

The patient was discharged the day after the procedure. Before discharge, we confirmed acceptable device parameters (threshold of 0.38 V at 0.24 milliseconds, impedance of 830 Ω) and verified proper LPM positioning radiographically (Figure 4E). At the 4-month follow-up, the device parameters remained stable. Unfortunately, the patient’s condition later deteriorated as a result of heart failure exacerbated by pyelonephritis, ultimately leading to her death.

## Conclusions

Our case highlights that the implantation of a third LPM is a viable option for patients with complex cardiac histories, including patients with previous device infection, hypertrophic cardiomyopathy, PTSMA, and immunocompromised status. Although the preferred approach often involves replacing a failed device following its extraction, our case demonstrates that implantation of a third LPM can be both possible and feasible when there is a high risk of extraction-associated complications in frail patients. Additionally, this case underscores the importance of careful fluoroscopic technique and heightened vigilance during multiple LPM implantations, particularly in visualizing device positioning and confirming proper fixation. These technical considerations are crucial for ensuring successful outcomes in complex cases involving multiple LPMs.

## Funding Support and Author Disclosures

This research has received no specific grant from public, commercial, or not-for-profit funding agencies. Dr Yagasaki has received MitraClip proctoring fees from Abbott, and although they are not directly related to this article, Abbott produces devices that could potentially compete with the Micra device. Dr Warita has received Micra proctorship fees from Medtronic Japan. Drs Suzuki and Noda have reported that they have no relationships relevant to the contents of this paper to disclose.

## Appendix

Video 1 Second LPM Implantation

Video 2 During and After Tether Removal

Video 3 Forward Movement of the Second LPM

Video 4 Third LPM Implantation
